# Gamification in EFL/ESL instruction: A systematic review of empirical research

**DOI:** 10.3389/fpsyg.2022.1030790

**Published:** 2023-01-05

**Authors:** Songcun Zhang, Zuwati Hasim

**Affiliations:** Faculty of Education, University of Malaya, Kuala Lumpur, Malaysia

**Keywords:** gamification, language learning, EFL/ESL instruction, feedback, gamification elements, learning effects, student motivation

## Abstract

**Introduction:**

This systematic review aims to present the characteristics of the recent research in gamified EFL/ESL instruction, benefits and drawbacks of using gamification in EFL/ESL instruction, and gamification elements.

**Methods:**

The researchers carried out database search in both Web of Science and the Scopus for relevant articles using 15 related key terms. Finally, forty journal articles aligned with the inclusion criteria.

**Results:**

The results found that gamification has been widely utilized in more than ten non-English-speaking countries and various English language skills, which indicated that gamification has gained popularity in facilitating EFL/ESL learning. The benefits of using gamification included improving students’ English language skills and abilities, positively affecting students’ attitudes and emotional responses, providing an authentic language learning environment and cultivating students’ comprehensive competence. The drawbacks of using gamification mainly included the technical problems, short-lived positive effect, and the negative influence caused by the gamified competition, and so forth. The most frequently used gamification elements were feedback, points, quiz, digital badges, leaderboard, and reward, followed by progress bar, story-telling, challenge, videos, time limit, and competition.

**Discussion:**

The results provide a better understanding of the state of using gamification in EFL/ESL instruction in recent years. It will be useful for researchers seeking to understand and evaluate gamification as well as to practitioners interested in using gamification.

## 1. Introduction

Gamification is defined as the use of game design elements in non-game contexts, yet it did not attract widespread adoption until the second half of 2010 (Deterding et al., 2011). Werbach (2014) redefined gamification and considered it as the process of making non-game activities more game like. Gamification is also defined as the application of game design elements like points, leaderboards, and badges in a non-game context, to provide a game-like learning experience (Landers et al., 2017). Kapp (2012) considered that gamification was not simply the use of game mechanics and elements to make learning more engaging, but the idea of increasing learners’ engagement, creating interactive learning contexts, and achieving students’ learning autonomy. Badges, rewards, cumulative scores, and competitive scores seem to provide visible incentives for students and expected behaviors in education (Shortt et al., 2021). The design of gamified learning environment should combine three distinctive concepts: dynamics, mechanics, and components (Bicen and Kocakoyun, 2018). Game dynamics refer to status, reward, self-expression, competition under rules that are explicit and enforced, and achievement, and others. Game mechanics refer to level-system, narrative context, challenge, achievements, leaderboards, and the like (Bicen and Kocakoyun, 2018). Game components or elements include self-representation with avatars, feedback, points, trophies, badges, progress bar and virtual presents, and the like (Deterding et al., 2011). All these elements are intended to arouse participants’ feelings of interest, competitiveness, curiosity, and frustration, convince them, and even change their behaviors, so that a gamification application could facilitate their learning process. Gamification is easy to implement on portable mobile devices and widely applied in digital environment (Su et al., 2021), therefore, this study focuses on the digital gamification.

Recent years have witnessed the emergence and utilization of digital gamification in EFL/ESL teaching and learning (Dehghanzadeh et al., 2019). English as a foreign language (EFL) is a term used to describe the study of English as a foreign language in a non-English speaking country. English as a second language (ESL), also called English as an additional language, is the non-English native speakers’ study of English in a predominantly English-speaking country (Barber et al., 2009). This study puts the two terms together, because both EFL and ESL refer to English learners whose first language is not English. Given the challenges faced by non-native English speakers, how to improve students’ English listening, speaking, reading and writing abilities have become important research topics (Liu and Chu, 2010; Dehghanzadeh et al., 2019; Zohud, 2019).

There have been many teaching approaches throughout the history of EFL/ESL instruction. Since the 1970s, the label “communicative” began to be applied in EFL/ESL instruction, and the associated teaching approaches are communicative language teaching and task-based language teaching (Howatt and Smith, 2014). Communicative language teaching (CLT) is an approach that emphasizes interaction and communication in the process of language study (Savignon, 1991). However, some scholars argued that CLT has failed its intended goals in many EFL settings, because EFL contexts did not provide enough opportunities for students to use English outside of class time (Humphries and Burns, 2015; Lee and Wallace, 2018). As a subcategory of CLT, task-based language teaching focuses on the use of authentic language to complete meaningful tasks, such as conducting an interview, visiting a doctor, or planning an upcoming trip in the target language (Skehan, 2003). There are some other commonly employed teaching approaches in EFL/ESL instruction like the flipped learning, which aims to give teachers and students power to flip the traditional classroom: Students can learn lectures at home and spend their time at school doing homework (Hockly and Dudeney, 2018). Some studies found that the flipped learning was successful in achieving the instructional goals of the EFL class (Chen Hsieh et al., 2017); EFL learners in the flipped classroom achieved higher average scores than those in the non-flipped classroom (Lee and Wallace, 2018).

In the twenty-first century, with the development of revolutions in technology and education, there is an increasing variety of learning contexts to create new opportunities for language learners, such as “social media contexts, gaming platforms, collaborative- and telecollaborative-based projects, and numerous mashups” (Kessler, 2018, p. 208). Gaming is the increasingly popular domain (Kessler, 2018). Gamification has become an innovative trend in education which aims to make the learning process more attractive to students in a fun and humorous learning environment and is believed to facilitate and encourage students to participate in learning (Deterding et al., 2011; Lee and Hammer, 2011; Kapp, 2012; Bicen and Kocakoyun, 2018). In gamified learning contexts, students can feel a sense of engagement and enjoyment, receive immediate feedback, achieve success in striving against a challenge and overcoming it, finally have a sense of accomplishment (Bicen and Kocakoyun, 2018).

Given the increasing popularity of gamification in language learning, there appeared several reviews on gamification in teaching and learning languages (Dehghanzadeh et al., 2019; Dehganzadeh and Dehganzadeh, 2020; Shortt et al., 2021; Su et al., 2021). Su et al. (2021) compared 64 high-quality studies from January 2000 to August 2020 investigating mobile game-based language learning (MGBLL) and non-mobile game-based language learning (NMGBLL). This study found a wide application of gamification in language learning, followed by immersive games and simulation games, maybe because they possessed rich game elements like goals, continuous feedback, and control, which could maintain learners’ motivation and confidence and raise their curiosity. It also found that gamification was widely applied by MGBLL studies, maybe because gamification is easy to implement on portable mobile devices. The most commonly appearing game elements were “goals or rules, sensory stimuli, and adaptive challenges” (Su et al., 2021, p. 16). As for adaptive challenges, it means that a well-designed game is able to adapt challenges to match learners’ abilities so that tasks are not too easy or too difficult for learners. The most common learning outcomes were vocabulary acquisition and students’ positive affective states. Based on 35 articles from 2012 to 2020, Shortt et al. (2021) systematically reviewed the issues of design, application, and pedagogies in the use of Duolingo, a popular platform of gamification in Mobile-Assisted Language Learning applications. The findings of this study indicated a positive correlation between the use of Duolingo and foreign language performance, like improving academic achievement in English, improvement in English vocabulary, listening skills and English communicative skills. More importantly, participants highlighted the interactive gamified nature of Duolingo’s design, and some gamification elements were perceived positively, like badges and streaks, points and leader boards. However, once the novelty effect of gamified presentation wears off, “the gamification elements cannot compensate for the design decisions prioritizing competition over collaboration, repetition and translation over meaningful feedback and context” (Shortt et al., 2021, p. 22). Therefore, competition and repetition are not the necessary elements when designing gamified learning activities. Instead, elements like collaboration, meaningful feedback and context should be valued.

Similarly, Dehganzadeh and Dehganzadeh (2020) also found that Duolingo was the most frequently used gamified platform for language learning in recent years, providing playful opportunities for language learning. This study also examined the 28 articles selected from Web of Science, ERIC, and Scopus in terms of languages and found that English language was the most frequently used second language. This may be due to the fact that learning English language has been of major importance in many non-English speaking countries (Turan and Akdag-Cimen, 2020). Therefore, the utilization of gamification in EFL/ESL instruction is worthy of further exploration. Dehghanzadeh et al. (2019) conducted a systematic review of the use of gamification for ESL learners in digital environments through 22 publications from 2008 to 2019 and discovered that ESL learners’ experiences were positive in using gamification; the positive outcomes were related to engagement, motivation, and enjoyment. However, the researchers did not pay attention to the use of gamification among EFL learners. Given the large amount of EFL learners, their utilization of gamification, and the challenges of using digital gamification in EFL/ESL instruction, there is a need to conduct a systematic review of gamified EFL/ESL instruction and present an overview of the state of utilizing gamification for EFL/ESL learners.

There were contradictory research findings on the impact of gamification on EFL/ESL instruction and learning. A plethora of studies has reported and confirmed the potential benefits of employing gamification by EFL/ESL learners, these include reducing students’ English learning anxiety (Hwang et al., 2017; Hung, 2018; Barcomb and Cardoso, 2020); increasing students’ learning interest, motivation, and engagement (Hwang et al., 2017; Bicen and Kocakoyun, 2018; Zohud, 2019; Reynolds and Taylor, 2020; Zou, 2020; Almusharraf, 2021); improving students’ learning performance (Wu et al., 2014; Hwang et al., 2017; Ling et al., 2019; Zohud, 2019; Barcomb and Cardoso, 2020); and fostering learners’ autonomy (Zohud, 2019; Setiawan and Wiedarti, 2020; Zou, 2020). In contrast, some other studies concluded that students who had access to the gamified content performed better than the control group in the short run; however, it had no effect on the students’ final learning outcomes (Domínguez et al., 2013; Calvo-Ferrer, 2017). Gamification is a double-edged sword, for students who are bored and do not wish to learn, rewards, and incentives might increase their learning motivation, while for students who are already motivated to learn, gamified learning activities might harm their intrinsic motivation (Hanus and Fox, 2015). In addition, some students were not able to use a gamified learning application, due to the Internet connection problems, the high pace of the game, its competitive nature, and the lack of detailed explanation after the game (Ebadi et al., 2021). The contradictory results on the influence of gamification on students’ motivation, satisfaction, empowerment, and achievement scores are worth further research. Therefore, there is a need to investigate the impact of gamification on EFL/ESL learners through a systematic review of the relevant empirical studies.

In addition, it is important for EFL/ESL teachers and researchers to know the existing gaming platforms and gamification elements to create digital gamified language learning activities for students. That is, English teachers should be equipped by digital literacy. Using digital technology to create gamified language learning activities is part of English teachers’ digital literacy. Unfortunately, teacher preparation for technology use in language education has often been neglected (Kessler, 2018); studies that address the digital literacies in ESL are worthy of further attention (Soyoof et al., 2021).

Driven by the aforementioned research gaps in current literature, this article reviews empirical research on using gamification in EFL/ESL instruction with a focus on the state of the art in utilization of digital gamification for EFL/ESL learners, the benefits and drawbacks of using gamification in EFL/ESL instruction, the main platform and gamification elements used in designing EFL/ESL learning activities. The results provide a better understanding of the state of using gamification in EFL/ESL instruction in recent years. It will be useful for researchers seeking to understand and evaluate gamification as well as to practitioners interested in using gamification in formal and informal learning environments.

Specifically, the present study addressed the following three research questions:

1). What are the characteristics of the current research into the use of gamification in EFL/ESL instruction in the selected studies?
2). What is the impact of utilizing gamification in EFL/ESL instruction in the selected studies?
3). What are the main gamification elements used in designing EFL/ESL learning activities in the selected studies?

## 2. Methods

### 2.1. Research design

**FIGURE 1:**
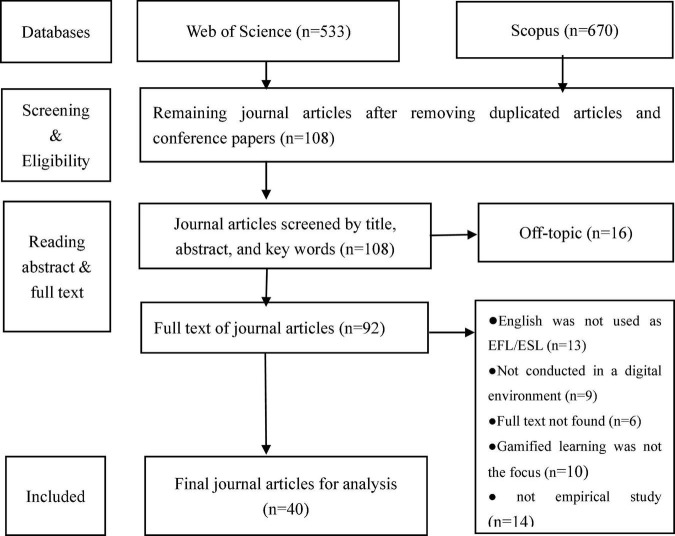
Flowchart process selection.

This study is a systematic review, which is defined as a comprehensive summary of the relevant research on given research questions through the process of identifying, selecting, synthesizing, and appraising all high-quality evidence (Grant and Booth, 2009). The aim of a systematic review is to answer research questions across a relatively narrow range of quality assessed studies (Soyoof et al., 2021). As such, the present systematic review investigated and selected journal articles related to the use of gamification in EFL/ESL instruction and learning, and carried out an analysis and discussion of the results based on the three research questions. This systematic review was guided by the Preferred Reporting Items for Systematic Reviews and Meta-Analyses (PRISMA) statement, which was an evidence-based set of recommendations to encourage transparent and complete reporting of systematic reviews and facilitate researchers to prepare and report an array of systematic reviews or meta-analyses (Sarkis-Onofre et al., 2021). All the procedure has been reflected in the flow diagram based on the PRISMA statement (See Figure 1).

### 2.2. Database and search strategy

To identify potential publications to be included in this review, literature was searched from the database of the Web of Science and the Scopus, with peer reviewed checked. As for the timeframe, there is no time limit for the publication date, which enables the researchers to showcase a holistic picture of the development and application of gamification in EFL/ESL instruction. The search string used in this study was (gamification OR gaming OR gamified OR game-based) AND (EFL OR ESL OR L2 OR English OR Foreign Language OR Second Language) AND (instruction OR teaching OR learning OR e-learning OR education). All the publications whose titles, abstracts, or keywords met the search strings had been taken into consideration, to collect the most relevant literature and select the high-quality journal articles in the field.

To refine the results and select high-quality publications, four inclusion criteria were used: (1) articles were published in English, because English, as a lingua franca, is used as a medium of communication among non-native speakers and native speakers (Taguchi and Ishihara, 2018); (2) the study focused on the use of digital gamification to support EFL/ESL teaching and learning, excluding educational games, video games, and serious games, because they are different terms (Kapp, 2012). Only choosing gamification-related articles is to make the review manageable and applicable to the three research questions; (3) articles were published in a peer-reviewed journal, excluding conference papers, book chapters, unpublished thesis, literature reviews, and secondary data analysis, because this systematic review searches for original studies which had been published and passed the rigorous editorial review; (4) the study was empirical research, to meet the overall design and objectives of this review.

The researchers screened the title, abstract and key words and decided whether the paper should be included in the data analysis based on the pre-defined inclusion criteria. If it was difficult to make the decision, the full-text version of the paper was read, then a further decision was made.

The initial search with the key words noted yielded 533 journal articles in the database of Web of Science and 670 journal articles in the database of Scopus. After removing the duplicated journal articles and conference papers from further analysis, 108 articles remained. The titles, abstracts, key words, and methodology of each article were read to further eliminate those publications that were not within the scope of our research. Finally, forty journal articles were included in this systematic review. Figure 1 shows the selection process of publications for the present study.

The selected publications were coded by referring to the technology-based learning review (TLR) model suggested by Hsu et al. (2012). To investigate the trends in technology-based learning, the dimensions of “Research purposes,” “Application domains,” and “sample groups” should be taken into account. Besides, the factors associating the three dimensions should also be included, such as “Research issues,” “Research methods,” and “adopted technologies/learning environments” (Hsu et al., 2012). We also referred to the coding schemes devised by previous scholars to analyze the target studies (Chang and Hwang, 2019; Dehghanzadeh et al., 2019). Eleven categories were adopted in this study to answer the three research questions: authors; publication source; learning environments; educational level; methodology; data collection method; experimental or not; gamification elements; benefits; content language learning; and research location.

## 3. Findings

### 3.1. Current research state of the use of gamification in EFL/ESL field

Supplementary Table 1 provides an overview of the empirical studies included related to the use of digital gamification in the EFL/ESL field.

Based on Supplementary Table 1, most of these journal articles were published in the last 6 years, which indicates that gamified EFL/ESL instruction is a relatively new field of research. Researchers of the studies reviewed used a variety of digital learning environments, not only including the widely used Kahoot (Hung, 2017; Zou, 2020; Alawadhi and Abu-Ayyash, 2021; Almusharraf, 2021; Chen, 2021; Ebadi et al., 2021), Duolingo (Bahjet Essa Ahmed, 2016; Guaqueta and Castro-Garces, 2018; Ajisoko, 2020), Moodle (Barcomb and Cardoso, 2020; Ho, 2020; Qiao et al., 2022), and some gamified English learning APPs used in China like Baicizhan (Dindar et al., 2021) and Shanbay (Fan and Wang, 2020); but also self-designed gamified software or webpage (Hwang et al., 2017; Hung, 2018), which indicates that digital gamified tools could play a vital role in EFL/ESL instruction and learning. The publication sources are influential SSCI-indexed or Scopus-indexed journals and to some extent show the characteristics of the combination of modern technology and language learning. The distribution of educational level is higher education (55%), secondary school (32.5%), and elementary school (12.5%). Most of these are experimental. Quantitative, qualitative, quasi-experimental, and mixed methods have been used for the research of gamification in the EFL/ESL field. Various data collection methods are used: questionnaires, class observations, interviews, researcher journals, students’ self-reflections, checklists, pre- and post-tests, and so forth. The content of language learning involves the instruction and learning of English vocabulary (27.6%), grammar (20%), speaking (15%), reading (15%), writing (10%), college English (7.5%), listening (7.5%), morphological awareness (2.5%), literature (2.5%), and business English (2.5%). Some of these studies have combined several aspects of learning content. For example, Fan and Wang (2020) investigated tertiary students’ EFL learning in vocabulary, reading, and speaking in China. These empirical studies were conducted in more than ten EFL/ESL countries: China, Malaysia, UAE, Korea, Netherlands, Saudi Arabia, Iran, Spain, Turkey, Singapore, Japan, Indonesia, and Ecuador, and so forth.

### 3.2. Learners’ experiences and the impact of gamification on learners

As shown in Supplementary Table 1, although there were some drawbacks of using gamification, many empirical studies reviewed reported that both students and teachers held a positive attitude toward using gamification in EFL/ESL learning and teaching, because a gamified course system did increase students’ motivation to learn (Liu and Chu, 2010; Hwang et al., 2017; Ge, 2018; Homer et al., 2018; Hung, 2018; Sun and Hsieh, 2018; Ho, 2020; Zou, 2020; Alawadhi and Abu-Ayyash, 2021; Almusharraf, 2021; Chen, 2021; Kaban and Karadeniz, 2021; Qiao et al., 2022), stimulate students’ interest and engagement in learning English (Hung, 2018; Kingsley and Grabner-Hagen, 2018; Sun and Hsieh, 2018; Ho, 2020; Zou, 2020; Alawadhi and Abu-Ayyash, 2021; Wang et al., 2021), help to create an authentic language environment (Wu et al., 2014; Mei and Yang, 2019), help students to make improvements in English skills performance and competence (Sandberg et al., 2014; Hung, 2017; Hwang et al., 2017; Sevilla Pavón and Haba Osca, 2017; Lam et al., 2018; Hashim et al., 2019; Hong et al., 2020; Zou, 2020), foster the habit of self-learning and realize learning autonomy (Sandberg et al., 2014; Rueckert et al., 2020), and help students to get better knowledge retention (Ge, 2018; Chen et al., 2019).

For example, Zou (2020) explored primary students’ and teachers’ perceptions of gamified flipped classroom in EFL context in China. A 1-year project was conducted among 277 primary students and eight teachers in Hong Kong. Kahoot and Edpuzzle were used as platforms to organize the English learning activities and allow participants to complete exercises, get immediate feedback, and a rank of students’ scores was to encourage students to perform better. The relevant data was collected via interviews, in-class observations, teachers’ and students’ self-reflections, and the researchers’ observation logs. The result revealed that both teachers and students believed that gamified flipped classrooms were beneficial. Students considered gamified classroom motivative, engaging, effective, and worthwhile, although it was to some extent challengeable. Teachers considered that it was effective in increasing students’ learning motivation and engagement, developing their learning skills and confidence, and improving their learning performance and outcomes. The implication was that the gamified flipped classroom led to development of primary students’ English learning motivation, confidence, and self-regulated learning skills. Therefore, it is suggestive to continue the gamified flipped EFL classroom among primary students.

Almusharraf (2021) explored undergraduate students’ perceptions of the impact of Kahoot on increasing engagement and classroom dynamics while reviewing writing structure, terminology, and knowledge in EFL online English literature courses in Saudi Arabia. There were sixteen literature classes in one semester, eight were traditional sessions with teacher-led lecturing and reading from the PowerPoint slides, and the other eight used Kahoot as a tool to review something previously taught and do formal assessment. Through an online survey and the classroom observations, it was found that the students’ engagement level was higher in game-based sessions. The findings also revealed that students showed favorable attitudes toward a game-based learning environment. Game-based sessions had a positive influence on student motivation and improved classroom dynamics, compared with the control group. All participants in the experimental group benefited from the game-based setting, and no significant differences in EFL learners’ perceptions of Kahoot were discovered among participants of different age groups and gender.

However, gamification is not a perfect approach. According to Supplementary Table 1, the drawbacks of utilizing gamification involve the technical problems (Hung, 2017; Guaqueta and Castro-Garces, 2018; Tan, 2018; Ebadi et al., 2021), fixed learning routines (Fu et al., 2021), the potential useless implementation of the gaming elements in teaching and learning (Iaremenko, 2017), not necessarily to improve students’ English skills (Homer et al., 2018; Lam et al., 2018; Qiao et al., 2022), and causing students’ high-level learning anxiety in a competitive learning context (Hwang et al., 2017; Ge, 2018). Moreover, some gamified elements like the leaderboard and the competition context might scare off some children and could not improve their academic performance (Li and Chu, 2021). In addition, once the novelty of gamification has worn off, the positive influence of gamification might be short-lived (Hung, 2018).

Overall, the teachers’ perspectives on using gamification in EFL/ESL courses showed some contradictory views (Krishnan et al., 2021; Luo et al., 2021). On one hand, Krishnan et al. (2021) concluded that it was beneficial for EFL teachers to use gamification, because it could aid the English language teachers and provide a wider toolkit for designing teaching activities. Gamification elements such as leader boards, progress bars, badges, and points could increase students’ engagement and motivation, help the learners become aware of how far they advanced in the level, and promote growth by encouraging healthy competition and collaboration. On the other hand, Luo et al. (2021) investigated the factors that influence teachers’ intention to use gamification in secondary schools in China and found that these EFL teachers showed negative attitudes toward gamification. These secondary school teachers in China were dubious about the capacity of gamification in providing learning opportunities, and it weakening of pedagogical purposes and teaching efficiency. They would also feel at risk of decreasing students’ scores in exams if using gamification. Finally, the technical feature of the tool is also a problem. Both the teachers and the students prefer tools with interface aesthetics and ease-to-use features. The aforementioned two studies hold different views about using gamification in EFL/ESL course, mainly because the educational levels of students and their learning purposes are different. The participants of Krishnan et al. (2021)’s study did not have the pressure of gaining a high score in high-stakes exams, but Luo et al. (2021) observed the high school students in China, who are expected to take part in College Entrance Examination. Gaining a high score in English is such an important thing which will determine their life. They cannot risk of failing in the College Entrance Examination.

### 3.3. Gamification elements used in the reviewed studies

A variety of digital gamification elements used in the reviewed publications have been presented in Table 1. The most frequently used elements were feedback, points, quiz, digital badges, leaderboard, and reward, followed by progress bar, story-telling, challenge, videos, time limit, and competition. The relevant less frequently used elements were avatars, collaboration, role playing, and QR code, and so forth. The gamified EFL/ESL learning system would usually be points-based or a levels system with some gamification elements like leaderboard, progress bar, avatars, badges, and feedback. Rewards would usually be given to participants if they presented correct answers, because appropriate reward strategies can elicit a better learning outcome on EFL learners (Ge, 2018).

**TABLE 1:** The gamification elements used in the publications reviewed.

Gamification elements	Frequency (N)	Gamification elements	Frequency (N)
Feedback	16	Points	15
Quiz	15	Digital badges	9
Leaderboard	9	Reward	7
Progress bar	5	Story-telling	5
Challenge	5	Videos	4
Time limit	4	Competition	4
Avatar	3	Role playing	3
QR code	3	Collaboration	2

For example, Lam et al. (2018) investigated the effects of digital game mechanics on secondary school students’ argumentative writing in Hong Kong. The experiment consisted of three groups and lasted for 7 weeks. The control group utilized traditional teacher-led direct-instruction approach in which the teacher taught students the key components of argumentation, the assessment rubric, and the writing strategies. Edmodo was used by the two experimental groups to post argumentative topics and the interaction among teachers and students. Experimental Group 1 utilized the blended learning and gamification approach, Experimental Group 2 used only the blended learning approach in which online educational materials and traditional teacher-led classroom methods were combined. The digital game mechanics of Experimental Group One included a points-based system in which students were awarded one point when they provided correct answers relevant to the topic and a leaderboard which was a digital score table that ranked students according to the points they earned and was refreshed every 2 weeks. Based on students’ interview data and their online Edmodo postings, the use of gamification elements motivated students to post significantly more messages on Edmodo and further increase their on-topic online contribution.

## 4. Discussions

This article aims to provide readers with the characteristics of the use of digital gamification in EFL/ESL empirical research, the probable benefits and drawbacks of using gamification on EFL/ESL instruction, and which gamification elements were used in designing and facilitating gamified learning activities in the selected studies. Forty SSCI-indexed or Scopus-indexed journal articles were selected with relevant key words, reviewed and analyzed by the researchers from different perspectives.

The findings are that the use of gamification has proliferated and gained popularity in EFL/ESL field. It has been utilized in many empirical studies in more than ten non-English-speaking countries. Unlike some previous studies (Phuong, 2020; Govender and Arnedo-Moreno, 2021) which claimed that online gamification was mainly used to teach vocabulary, and very little used to teach content knowledge and English grammar, not to mention other aspects of English learning, the quantitative findings of this study were that gamification could be widely used in teaching vocabulary, grammar, listening, speaking, reading, writing, pronunciation, college English, and even English literature. This indicates the feasibility and practicality of using gamification in actual EFL/ESL classrooms. Our review also found that gamification has been employed in different educational levels ranging from primary school to higher education, but being favored for the higher education.

In addition, gamification can be applied and combined with diverse learning environments, like the flipped classroom (Hung, 2018; Zou, 2020) and the ubiquitous learning environment (Liu and Chu, 2010) which is an everyday learning environment that is supported by mobile or embedded computers and wireless networks in our everyday life and aims to make learning happen anytime and anywhere (Ogata, 2009). In the flipped classroom, the pre-class self-learning could help students remember and understand the basic knowledge so that more time could be spent in class on gamified and interactive activities aimed at assisting students in applying, analyzing, and evaluating their knowledge. In the ubiquitous learning environment, students could be assigned to play a ubiquitous learning game in which they used cellphones to practice English listening and speaking during their free time, to perform a treasure hunt game outdoors during class time, and to collaboratively perform a story relay race in an actual context during class time. Students could learn English without the constraints of time and place. Overall, the combination of the advantages of gamified learning and flipped classroom or the ubiquitous learning environment could develop innovative approaches for language learners, help to create an effective learning process, and produce better learning achievement.

Although gamification has gained popularity in the EFL/ESL field, not all teachers consider that using gamification is acceptable and efficient in EFL/ESL contexts. For example, in a secondary school English class in China, teachers dare not risk using gamification (Luo et al., 2021), because they are afraid that gamification cannot help students get a high score in College Entrance Examination which is the most important summative assessment for Chinese secondary school students and determines whether students could be accepted by a university. To get a high score in this high stakes examination, all the curriculum and pedagogy in secondary schools serve for the College Entrance Examination. Students are required to complete many sets of model test papers; English courses aim at cultivating students’ linguistic knowledge and examination skills to get a high score. Based on the alignment theory proposed by Pickering and Garrod (2004), successful communication occurs when interlocutors well align their linguistic representation during dialogue and construct similar situation models to each other. Alignment of the situation model is achieved by lexical and syntactic alignment, that is, the alignment of information states rather than information transfer (Pickering and Garrod, 2006). Similarly, students would successfully get a high score in College Entrance Examination when they could well align their linguistic knowledge with the real test papers. Such kind of alignment would be achieved when students received enough training in completing examination papers. Accordingly, the English curriculum, pedagogy, and assessment have been well aligned and examination-oriented in secondary schools in China. However, gamification used in EFL/ESL instruction only provides information transfer which means delivering learning materials to students, not well aligned with the requirement of College Entrance Examination. Therefore, whether to use gamification depends on the learning purpose and demand.

The benefits of using gamification in EFL/ESL field can be analyzed from three aspects. Firstly, it was found that the gamified learning environment was beneficial for EFL/ESL learners in improving their English skills in listening, speaking, reading, and writing, compared with the conventional learning environments. It is necessary for schools and teachers to accommodate students with dynamic learning materials and impart knowledge to students effectively. Gamification was a useful approach to provide dynamic materials. It can be used not only as a study and teaching tool (Kaban and Karadeniz, 2021) but also as a review and assessment tool (Almusharraf, 2021; Chen, 2021). Consequently, it can improve students’ English competence and foster the habit of self-learning.

Secondly, gamification can positively affect EFL/ESL learners’ attitudes and emotional responses of interest, motivation, anxiety, and a sense of achievement. Enhancing students’ learning motivation is one of the most frequently reported positive learning outcomes in the articles reviewed. Chen (2021) investigated EFL learners’ views on lessons which integrated the assessment function of Kahoot and collaboration of Padlet. Based on the data from the student questionnaire, the findings of this research indicated that students considered the gamification approach to be novel, interesting, game-like, and contributive to English learning, and promote their interest and engagement in learning English, as well as their involvement and enthusiasm. These are all strategies used to promote learners’ motivation which would lead to students’ engagement and is the primary driver for successful EFL/ESL learning.

Thirdly, gamification was helpful in providing an authentic language learning environment and cultivating students’ comprehensive literacy. Since it was reported that Taiwanese EFL learners had a low-level genuine communication ability, Wu et al. (2014) examined the effect of using digital board games designed for Taiwan EFL classroom learning, searched for an optimal language learning experience, and investigated whether students’ communicative skills and intrinsic motivation would be improved through seeing relevant context of the language and receiving appropriate practice through gaming. The media of the digital board games included a board, illustrated cards, and some game pieces. The gamified board provided situational plots that demonstrated authentic language use and were presented in text form or as game actions. Graphics, rules, thematic descriptions, and game pieces were used to create an immersive gaming environment. The experiment was conducted at a senior high school in Taiwan, and the participants were ninety-six students divided into three groups: a normal instruction group, a digital board game language-learning group, and a board game language-learning group. Through comparing participants’ learning performance using a speaking test, the result was that the digital board game learning group could get a higher speaking performance and better communication ability. The digital learning playground was helpful to encourage students in speaking English. Therefore, the researcher reported that the use of gamification had provided a genuine language environment through digital gamified situational plots, improved students’ communication ability, and strongly suggested that the game blending learning should be given importance, and attached and integrated into schools’ curricula. Similarly, Chen (2021) also confirmed that gamification could provide a more enjoyable learning environment and collaborative opportunities, create interactive atmosphere, increase students’ engagement and develop their communicative abilities.

Overall, these empirical studies confirmed the benefits of using gamification in EFL/ESL instruction and learning. The learning outcomes of their experiment could include engagement, motivation, and reducing anxiety in English listening and speaking (Hung, 2018), or motivation, linguistic, digital, and intercultural skills and competence (Sevilla Pavón and Haba Osca, 2017). Therefore, it is suggested a continuation of the gamified EFL/ESL classroom among learners, explaining the purposes and implementation of gamified classroom explicitly to the students, and facilitating their good use of the gamified classroom. However, there are also some drawbacks about the utilization of gamification as mentioned previously, which can give implications for designing gamified learning activities. Next, attention needs to be paid on how to use gamification in EFL/ESL classrooms in a way that expands opportunities for learners to practice and improve English skills without exhausting the allotted time.

As Bicen and Kocakoyun (2018) noted, the design of gamified learning environment should combine three distinctive concepts: dynamics, mechanics, and components. Game dynamics used in the reviewed studies refer to reward, competition, collaboration, and quiz, and others. Game mechanics in the reviewed studies refer to level-system, challenge, leaderboards, and the like. Game components in the reviewed studies involve feedback, points, trophies, badges, progress bar, story-telling, and avatars, and the like. To have a positive impact on learning outcomes, various gamification elements have been used to design fair rules, clear goals and social opportunities in gamified English learning activities (Aldemir et al., 2018). This study found that feedback, points, quiz, and digital badges are the most popular gamification elements used in EFL/ESL instruction. Points and badges can give each activity a value, send supportive messages, help students to self-assess, and increase their engagement in activities (Castillo-Cuesta, 2020; Kaban, 2021; Khalilian et al., 2021). Therefore, as a rewarding strategy, collecting points from their participation should be applied to gamified learning environment continuously and systematically. Immediate feedback and quizzes can help students to become aware of their performance and progress (Tan, 2018; Lee and Park, 2020; Rueckert et al., 2020). Because teachers or peers could read students’ answers as soon as they are posted in the online gamified platform and give immediate feedback, students could evaluate their work in time and make improvements. A progress bar is also effective to provide clear goals and guidelines to participants. In Ding and Orey’s (2018) study, the progress bar used in their gamified learning activities displays students’ current scores, the average score of the class, and the awards. With the help of the progress bar, students are able to monitor their own progress and the class average, and see how many scores they need to earn the award, so that they are motivated to work harder if they are not the top students.

The next key points in designing gamified learning activities are challenge and competition, such as time limit and leaderboard. The leaderboard section lists participants based on their achievements, promotes the recognition of the top students’ achievements, and can provide a sense of competence (Ding and Orey, 2018). As noted by Lam et al. (2018), according to social comparison theory, human beings like to evaluate their competence and achievement by comparison with those of others (Festinger, 1954). Accordingly, using a leaderboard caters to the competitive and comparative nature of human beings which facilitates and prompts the productivity of students.

However, there are both merits and demerits in the application of competition. According to Li and Chu (2021), the leaderboards and the competition might scare off some children, made them lack self-confidence to demonstrate their mastery in the gamified English reading, and did not like reading. Therefore, it is recommended to add gaming elements like collaboration to the gamified learning activities, so that children who do not like competition can engage in it. Similarly, Dindar et al. (2021) investigated the effects of gamified competition and gamified cooperation in facilitating Chinese students’ English vocabulary learning. Participants were divided into a gamified cooperation group and a gamified competition group. All participants were asked to learn 20 new English words per day. In the gamified competition condition, if participants could study all the required words, they would receive 20 points at the end of the day and were presented with a leaderboard that showed their ranking in the group. In the gamified cooperation condition, if a participant studied all the required words, the group he or she belonged to received 20 points. The group points were doubled for that day, if all the group members finished their daily task. Groups in the gamified cooperation condition received different badges depending on their total group score. The research found that gamified cooperation and competition had similar impacts on task effort, learning achievement, and motivation. However, social relatedness in the gamified cooperation group was higher than in the gamified competition group. Future gamified learning activities design should explore ways to increase students’ motivation and performance through gamified cooperation.

Therefore, both Dindar et al. (2021) and Li and Chu (2021) recommended using collaboration in designing gamified learning activities rather than competition to facilitate students’ EFL/ESL learning. Shortt et al. (2021) also considered that collaboration and meaningful feedback were more feasible gamification elements when designing gamified learning activities, comparing with competition and repetition.

## 5. Conclusion, implications, and limitations

This study provides an overview of the current state of gamification use for EFL/ESL instruction and learning. Through reviewing 40 selected empirical studies, this research found that the use of gamification has proliferated in the EFL/ESL instruction and learning. The reasons for using gamification in EFL/ESL instruction include improving students’ English language skills and abilities, positively affecting students’ attitudes and emotional responses, providing an authentic language learning environment and cultivating students’ comprehensive competence. Based on Supplementary Table 1, its advantages outweigh its disadvantages. As implementation of gamification is increasingly permeating educational settings, it is critical to investigate how teachers can use gamification as tools in aiding EFL/ESL instruction inside and outside the classroom and become accustomed to the modern technology. This review study shows that the design of a gamified learning environment should consider three concepts: dynamics, mechanics, and components. The gamification learning designers should make a reasonable combination of game elements and students’ learning content and take students’ educational level, their cognition and capability into consideration.

Given the confirmed value of employing gamification for EFL/ESL instruction and learning, this review study strongly advises the exploration of various game-based learning applications to develop English language skills for EFL/ESL learners. This review contributes to a growing body of knowledge on the utilization and effects of gamification in EFL/ESL area and is intended to provide researchers and teachers, who would become material designers of gamified learning materials and early adopters of the gamified classroom, with the information on how to design innovative gamified learning materials, refine the EFL/ESL learning process, and promote EFL/ESL learners’ productivity.

### 5.1. Implications

There are several implications for the future research. Most of the reviewed articles investigated the application of gamification from the students’ perspective. More research is needed to explore both teachers’ and schools’ perspectives. Do teachers have enough knowledge and confidence in employing gamification in their classroom effectively? How many teachers actually used gamification in their classroom? What factors would affect teachers’ use of gamification? Do schools have enough and appropriate gamification learning tools for EFL/ESL instruction and learning? How should schools prepare for appropriate gamification learning tools to facilitate EFL/ESL instruction? These are all important issues related to the application of gamification in the EFL/ESL field. As previously mentioned, the distribution of educational level in the studies reviewed is higher education (55%), secondary school (32.5%), and elementary school (12.5%). Future research could focus more on the utilization of gamification in elementary school and kindergarten stages. What kind of guidance do children need in applying gamification in English learning? How do individual student factors influence their learning behaviors and outcomes in gamified classrooms, factors such as age, gender, prior knowledge, English proficiency, and game proficiency? In addition, this study calls for serious attention from EFL/ESL education policy-makers, there a need to take the utilization of gamification in EFL/ESL classrooms into consideration since most of the selected studies confirmed the benefits of using gamification. The favorable policies chosen today will influence how EFL/ESL education develops tomorrow. Utilizing gamification in EFL/ESL education may bring positive changes for tomorrow.

### 5.2. Limitations

There are several limitations in this study. First, this study only covers the related SSCI-indexed and Scopus-indexed journal publications, which might lead to the deficiencies in the literature. Second, this study excludes conference papers, book chapters, unpublished thesis, literature reviews, and secondary data analysis in the selection of the publications, so that it would be manageable, avoiding too much literature. Although the researchers of this study tried their best to select high-quality and representative relevant empirical studies, some good articles published in the scope of exclusion criteria may still have been missed.

## Data availability statement

The original contributions presented in this study are included in the article/Supplementary material, further inquiries can be directed to the corresponding authors.

## Author contributions

ZH contributed to the design of the research and proofread the manuscript. SZ contributed to collect the data, performed the analysis, and wrote the manuscript. Both authors contributed to the article and approved the submitted version.
