# Supplementary material for: Gamification in EFL/ESL instruction: A systematic review of empirical research
Source: Front Psychol. 2023 Jan 5;13:1030790. doi: 10.3389/fpsyg.2022.1030790 (PMC9849815; doi:10.3389/fpsyg.2022.1030790)
Supplement: Supplementary file 1 [file Table_1.pdf]

Appendix A. Table 1. Quantitative description of the reviewed empirical studies.

Authors	Publication source	Learning environment	Educational level	Methodology	Data collection method	Experi mental	Gamification elements	Benefits	Drawbacks	Content language learning	Research location
Ajisoko (2020)	International Journal of Emerging Technologies in Learning	Duolingo	higher education	mixed method	tests, questionnaire	Yes	points, quiz,	facilitating vocabulary learning, students' positive responses to gamification		vocabulary	Indonesia
Alawadhi & Abu-Ayyash (2021)	Education and Information Technologies	Kahoot	higher education	mixed method	semi-structured interview, survey	Yes	quiz	students' positive attitude towards Kahoot, increased students' motivation, improved classroom engagement, and enhanced learning experience	not necessarily to improve students' exam scores	vocabulary, grammar items	UAE
Almusharraf (2021)	Interactive Learning Environments	Kahoot	higher education	mixed method	survey	Yes	challenge, quiz, group competition, feedback	improving students' engagement and classroom dynamics, can be used as a review tool		English literature	Saudi Arabia
Barcomb and Cardoso (2020)	Computer Assisted Language Instruction Consortium Journal	Moodle	secondary school	mixed method	tests, questionnaire, user logs, interview	Yes	leaderboard, avatar, points, badges, videos, quiz	reducing pronunciation anxiety, improving pronunciation		phonlogy	Japan
Castillo-Cuesta (2020)	International Journal of Emerging Technologies in Learning	Educaplay	higher education	mixed method	Pre and post-questionnaires, writing rubrics	Yes	cloze activities, crossword puzzles, matching tasks, and unscramble sentences, feedback	improvement in students' EFL writing, enhancing students' grammar and vocabulary knowledge		grammar, vocabulary	Ecuador
Chen et al., (2019)	ReCALL	PHONE Words	higher education	mixed method	questionnaire, pre-, post- and delayed posttest, log files of usage, interview	Yes	badges, quiz, sticker, word list, test	significantly improving students' vocabulary acquisition and retention, learners' positive attitude toward gamification		vocabulary	China

Chen (2021)	Education and Information Technologies	Kahoot, Padlet	higher education	qualitative	questionnaire	Yes	quiz, QR code, feedback	students' positive perceptions toward gamification, improving students' learning motivation and engagement	Internet connectivity	College English	China
Dindar et al., (2021)	British Journal of Educational Technology	Baicizhan, Wechat	higher education	quantitative	questionnaire, test,	Yes	points, leaderboard, badge, raffle, gift card	the positive influence of gamified cooperation on students' social relatedness	the ineffectiveness of gamified competition on students' social relatedness	vocabulary	China
Ebadi et al., (2021)	Interactive Learning Environments	Kahoot!	higher education	qualitative	open-ended questionnaires, semi-structured interviews	Yes		boosting students' interest in learning and their ambitions for success	Internet connection problems, the high pace and competitive nature of the game, the lack of detailed explanation after the game	grammar	Iran
Fan & Wang (2020)	Behaviour & Information Technology	Shanbay Word, Shanbay Reading, Shanby Colloquial Language	higher education	quantitative	questionnaires	No	role-play, badge, progress bar, words memorising player killing	positive effect on students' learning performance		vocabulary, reading, speaking	China
Fu et al. (2021)	International Journal of Emerging Technologies in Learning	gamified English vocabulary learning Apps	higher education	qualitative	interview	No		students' positive attitude towards gamified vocabulary learning, enhancing students' learning motivation and habits	fixed learning routines	vocabulary	China
Ge (2018)	Computers & Education	Rain Classroom	higher education	quantitative	pre-. post-, and delayed posttest, questionnaire	Yes	quiz, points, time limit, immediate feedback	better knowledge retention, stimulating learning motivation	a high-level learning anxiety	grammar	China
Guaqueta & Castro-Garces (2018)	English Language Teaching	Duolingo, Kahoot	secondary school	mixed method	Tests, survey, a research journal	Yes	quiz, feedback	fostering students' vocabulary building, providing more dynamic and fun way to learn	internet connectivity problems,	vocabulary	Colombia
Hashim et al. (2019)	Arab World English Journal	Socrative, PowerPoint Challenge Game, and Kahoot!	secondary school	quasi-experimental	pretest, posttest	Yes	quiz, challenge	effective in students' grammar achievement, motivation and fun		grammar	Malaysia

Ho (2020)	Innovation in Language Learning and Teaching	Sketchpad, Moodle	higher education	mixed method	questionnaires, interviews, and a writing test	Yes	digital drawing, story-telling, competition, guess	enhancing students' understanding of the narrative structure, benefiting collaborative learning and promoting social interactions in class		narrative writing	China
Homer et al. (2018)	Educational Technology & Society	ClassDojo	elementary school	mixed method	pre-and post-test, teacher observation, student survey,teacher reflection	Yes	digital badges and points, avatar	improving students' oral score, positive impact on students' motivation and behavior	no siginificant improvement in students' reading score	speaking and reading	China
Hong et al., (2020)	Computer Assisted Language Learning	TipOn	secondary school	quantatitive	questionnaires, pre-and post-test	Yes	gamified questions, rewards	positive efects on students' learning performance		grammar	China
Hung (2017))	Interactive Learning Environments	Kahoot	higher education	quasi-experimental	summative assessment, a perceptive survey, and student interview	Yes	quiz, points, time limit, leaderboard, nickname, sound effects	postive influences on student learning, allowing students to bring their own devices to access, bridging the pre-class and in-class activites of flipped learning lessons	possible logitstic or technical problems	vocabulary	China
Hung (2018)	ELT Journal	self-designed gamification webpage	higher education	mixed method	questionnaire, interview	Yes	videos, feedback, tokens, dice, QR-code	reducing students' anxiety about speaking English, enhancing their learning motivation	The positive effect of gamification might be short-lived once their novelty has worn off.	speaking and listening	China
Hwang et al., (2017)	Computers & Education	self-designed game	secondary school	quasi-experimental	pre- and posttests, questionnaire	Yes	battle, trigger points, coding result	improving students' learning motivation and learning achievement, benefiting the more anxious students	not decreasing students' English anxiety	listening	China
Iaremenko (2017)	Information Technologies and Learning Tools	Kahoot	higher education	qualitative	questionnaire	Yes	quiz, competition	enhancing students' learning intrinsic motivation and engagement	the potential useless implementation of the gaming elements in teaching and learning	grammar	Ukraine

Kaban (2021)	International Journal of Mobile and Blended Learning	gamified electronic reading platform	secondary school	mixed method	questionnaire, interview, teacher journals	Yes	quiz, feedback, progress bar,badges, leaderboard, customizing the avatar/environment, fixed reward schedule,	improvement of students' reading comprehension levels and more positive reading attitudes, instant and formative feedback		reading	Turkey
Kaban & Karadeniz (2021)	SAGE Open	Raz-Kids	elementary school	quasi-experimental	pre-and post-test, a reading motivation scale	Yes	timer, quiz	positive effect on students' reading motivation	need reliable and constant internet connection, no significant improvement in students' reading score	reading	Turkey
Khalilian et al. (2021)	The Journal of Language Teaching and Learning	Kahoot	secondary school	quasi-experimental	questionnaire, pre-and post grammar test	Yes	points, quiz, leaderboard, color and symbol	positive effect on learners' motivation and structural ability		grammar	Iran
Krishnan et al. (2021)	Sustainability	Classcraft	higher education	Design and Development Research	questionnaire	Yes	level system, avatar, reward, storyboard, quest, points, feedback	enhancing teachers' professional competency			Malaysia
Lam et al. (2018)	Language Learning & Technology	Edmodo	secondary school	qualitative	pre-and post-test written essays, students'online Edmodo postings, interview	Yes	points-based system, leaderboard, feedback, role-playing	increasing students' on-topic online contributions	fail to significantly improve students' argumentative writing	writing	China
Lee & Park (2020)	Computer Assisted Language Learning	a location-based AR app	higher education	mixed method	open-ended questions, reflection papers, scenes created by students,	Yes	progress bar, scores, feedback, role-playing	supporting students' affective, cognitive, and social domains		College English	Korea
Li and Chu (2020)	British Journal of Educational Technology	self-designed gamified e-learning platform	elementary school	mixed method	examination, interview, questionnaire, longitudinal tracking	Yes	leaderboard, points, battles, virtual badges	enhancing students' reading interest and motivation, improving students' reading abilities and academic performance, constructing an online social community and increase students' social interaction, helping students gain greater autonomy	The leaderboards and the competition scared off some children.	reading	China

Liu and Chu (2010)	Computers & Education	Handheld English Language Learning Organization	secondary school	mixed method	test, questionnaire, interview	Yes	virtual and real reward, story relay race, digital treasure hunt game	achieving a better learning outcomes and motivation		listening and speaking	China
Luo et al., (2021)	Education and Information Technologies		secondary school	mixed method	a survey with open questions, a semi-structured interview	No			teachers' negative attitudes and limited skills toward gamification		China
Mei and Yang (2019)	Sustainability	a mobile AR-based and environment -themed scavenger	higher education	mixed method	questionnaire, observatioin, interview, field notes	Yes	geolocation-based games, tours, and interactive stories	improving students' environmental knowledge and awareness, enriching students' language learning experience	the digital downsides (e.g., distraction and shortage of devices)	writing	China
Qiao et al., (2022)	British Journal of Educational Technology	Moodle	secondary school	mixed method	pre- and posttests, semi-structed interview	Yes	a point-based reward framework, leaderboard, progress bar, badges, and a gold trophy cup	enhancing students' cognitive and motivational outcomes	no siginificant effect on students' word reading, reading comprehension, and affective engagement	morphological awareness	China
Rueckert et al. (2020)	Foreign Language Annals	rezzly.com	higher education	mixed method	questionnaire, written reflections, teacher journal	Yes	badges, points, feedback, repetition	providing brain-friendly learning, promoting students' learning autonomy		College English	Ecuador
Sandberg et al., (2014)	Computers & Education	Self-designed application	elementary school	quantitative	pre- and post-test	Yes	quiz, feedback, challenges, rewards, storyline,	students' positive learning outcomes, more efficient learning	technique problems such as having a disturbing bug. Gamification does not motivate the children to spend more time on the learning material.	vocabulary	Netherlands
Sevilla Pavón & Haba Osca (2017)	Iberica		higher education	mixed method	questionnaires	Yes	points, performance graphs, quests, avatars, a reward system, feedback	improving students' linguistic, digital and intercultural ability and motivation, students' high level of engagement and positive attitude		Business English	Spain

Sun & Hsieh (2018)	Journal of Educational Technology & Society	gamified interactive response system	secondary school	mixed method	questionnaire, pre-and posttest	Yes	polling activities, challenge, clickers	improving the students' levels of intrinsic motivation, overall engagement, emotional engagement, and focused attention		English	China
Tan (2018)	Online Learning	The Protége	higher education	mixed method	questionnaire, pre-and post test	Yes	feedback, challenge	motivating and helping students in scaffolding reading materials	technical issues and narrative dissonance	reading	Singapore
Wang et al., (2021)	Sustainability	XploreRAFE +	higher education	qualitative	interview, observation, students' essays, video recordings	Yes	leaderboard, timer, bonus points,	enhancing students' piquing curiosity, optimal learning experience, and experiencing meaningfulness		English writing	China
Wu et al. (2014)	Educational Technology Research and Development	digital learning playground	secondary school	mixed method	test, interview, questionnaire	Yes	situational plots, illustrated cards, feedback	encouraging students to speak more, achieving a better communication ability		speaking	China
Zou (2020)	Journal of Computers in Education	Edpuzzle, Kahoot	elementary school	mixed method	observations, researcher journals, teachers and students' self-reflection, interviews	Yes	real-time feedback, videos, competitions, collaborations,	promoting students' motivation, engagement, developing learning skills and confidence, improving performance		vocabulary	China
